# Strengthening Intersectoral Collaboration for Primary Health Care in Developing Countries: Can the Health Sector Play Broader Roles?

**DOI:** 10.1155/2010/272896

**Published:** 2010-04-29

**Authors:** Omokhoa Adedayo Adeleye, Antoinette Ngozi Ofili

**Affiliations:** Department of Community Health, School of Medicine, University of Benin, Benin City, Nigeria

## Abstract

Many strategic challenges impeding the success of primary health care are rooted in weak strategic inputs, including intersectoral collaboration. Some encouraging evidence from programmes, projects, and studies suggests that intersectoral collaboration is feasible and useful. The strategy has the potential to fast-track the attainment of Millenium Development Goals. However, the strategy is not commonly utilised in developing countries. The health sector expects inputs from other sectors which may not necessarily subscribe to a shared responsibility for health improvement, whereas the public expects ‘‘health” from the health sector. Yet, the health sector rarely takes on initiatives in that direction. The sector is challenged to mobilise all stakeholders for intersectoral collaboration through advocacy and programming. Pilot projects are advised in order to allow for cumulative experience, incremental lessons and more supportive evidence.

## 1. Preamble

Thirty years after the 1978 Alma Ata Declaration, the Primary Health Care (PHC) goal to achieve “an acceptable level of health for all the people of the world by the year 2000” remains unmet and remote in many developing countries. The declaration envisaged the urgent application of a wide range of strategies including poverty reduction, literacy improvement, provision of basic health services and infrastructures, appropriate technology, intersectoral collaboration (ISC), community mobilisation and participation, a strong political will, economic and social development based on a New International Economic Order, and international cooperation to improve the health status of individuals, families, and communities [1]. These strategies are important determinants of the success of PHC. One of them, ISC, is the primary focus of this paper.

## 2. Defining Intersectoral Collaboration

In the health literature, the term *intersectoral collaboration* frequently refers to the collective actions involving more than one specialised agency, performing different roles for a common purpose. But the point must be made that *multisectoral actions* are necessary but not sufficient to constitute ISC. Thus, vertical but related multisectoral actions do not constitute ISC. The coordination of efforts of sectors as an essential requirement for ISC is highlighted in the 1978 Declaration of Alma Ata, Article VII (4):

*(PHC) involves, in addition to the health sector, all related sectors and aspects of national and community development, in particular agriculture, animal husbandry, food, industry, education, housing, public works, communications and other sectors; and demands the coordinated efforts of all those sectors;*

and Article VIII:

*All governments should formulate national policies, strategies and plans of action to launch, and sustain primary health care as part of a comprehensive national health system and in coordination with other sectors. To this end, it will be necessary to exercise political will, to mobilize the country's resources and to use available external resources rationally [1].*

More recently, the WHO promoted the concept of intersectoral action for health (IAH) as “a recognised relationship between part or parts of the health sector with parts of another sector which has been formed to take action on an issue to achieve health outcomes (or intermediate health outcomes) in a way that is more effective, efficient or sustainable than could be achieved by the health sector acting alone” [2]. Being a recognised relationship suggests that IAH is a managed process. The involvement of parts of sectors may be understood as pointing to the structural and functional nature of the relationship, not just a conceptual one. Improved effectiveness, efficiency and sustainability refer to the benefits expected from the relationship based on specified roles and responsibilities played. For the purpose of this article, IAH may conveniently be taken as synonymous with ISC for health. It must be borne in mind that the collaboration can be between different departments and bodies within the government, between actors within and outside government, such as civil society organisations, for-profit private organisations and communities; all the actors may be outside the government.

## 3. The Core Place of Intersectoral Collaboration in Primary Health Care

The WHO defines health as “a state of complete physical, mental and social wellbeing …” as presented in her constitution [3] and reechoed in the Alma Ata Declaration [1]. These broad aspects of wellbeing are well beyond what the health sector alone can handle.

Considered individually, the earlier stated strategies of PHC require a very wide range of inputs from many sectors. For example, literacy improvement is mainly the task of the education sector; developing appropriate technology is multisectoral but may require key inputs from the technology and industrial sectors; and poverty reduction will draw from strategic initiatives of the economic planning sectors for multisectoral implementation. Thus, PHC strategies fundamentally call for multisectoral inputs.

Some services normally require multidisciplinary and sometimes intersectoral inputs. For example, school health programs, which may engage health and education sectors, aim at improving the wellbeing of children, thus reducing school absenteeism and improving learning. School health services are common worldwide. At the international level, this concept was used to develop the Focussing Resources on Effective School Health (FRESH) initiative whose “goal is to improve learning and educational achievement by improving the health and nutritional status of school-age children. The FRESH partnership was developed by the World Bank, WHO, UNICEF and UNESCO …”. In the words of the WHO Director General on FRESH, “…without proper education, health suffers. And without proper health, good education is not possible. In this our work is linked and it depends on each other …”. Similarly, the UNESCO Director General said, “If the bodies of the learners are healthy, then their minds will be more receptive to learning. By ensuring the health and education of young people, you are offering them the strongest tool of all for the eradication of poverty” [4]. Again, the health sector often engages the information sector to support mass information and mobilisation efforts for mass immunisation programs as often practiced in Nigeria. 

 Overlaps are possible between the benefits derivable from multiple PHC strategies as illustrated in the preceding paragraph. In this regard, *provision of basic infrastructures* such as electricity is a PHC strategy that can contribute to *poverty reduction* by providing energy for the manufacturing sector which then generates employment and income. *Poverty reduction* is achievable through government-facilitated storage and marketing schemes for agricultural products from small and medium-scale farms. Such an initiative can economically empower a farming community to engage in infrastructural development partnership projects with the government (*community participation*). The improvement in social wellbeing derived from these strategies constitutes improvement in health and economic empowerment that may improve healthful behaviour and financial access to health services.

## 4. Primary Health Care, Millennium Development Goals and Intersectoral Collaboration

 The MDGs include the reduction of child death, improvement in maternal health, and combating HIV/AIDS, malaria and other major diseases. These all call for well-functioning health systems, but are highly dependent on inputs from other sectors. For example, optimal public power supply is required for the efficient maintenance of vaccine cold chains so as to maintain the potencies of vaccines to reduce child death and for blood banks to provide blood to save the lives of haemorrhaging women at delivery. Again, some other MDGs are also related to the health sector. For example, eradication of extreme poverty and hunger (associated with economic development and agricultural sectors) and ensuring a sustainable environment (associated with environmental sector) produce health benefits. At least three key points emerge from these observations. First, MDGs and their indicators are closely related to PHC tenets, as exemplified by interventions related to maternal and child health, water and environment and poverty eradication. Secondly, interventions developed in response to the Alma Ata Declaration on PHC more than 30 years ago were meant to be urgent, and the MDGs are time-bound and now urgent, having well past mid-term. Thus, MDGs appear to fast-track PHC, at least conceptually. Thirdly, while ISC *per se* is not formally presented as an MDG strategy, it is required in that regard as has been explained for PHC.

## 5. The Problem Statement

Despite the core place that ISC has in the implementation of PHC and the attainment of MDGs, this strategy (beyond vertical but related multisectoral actions) is not widely utilised in developing countries, including Nigeria. Yet opportunities exist to use this strategy to achieve health outcomes that, according to the WHO, are “more effective, efficient or sustainable than could be achieved by the health sector acting alone” [2]. 

In a more conceptual and practical sense, a “tripod of neglect” of ISC for health can be described. First, the “nonhealth” strategies (such as the provision of safe water) are outside the statutory control of the health sector. This is particularly important since the public expects “health”—including promotive, preventive, curative, and rehabilitative health services—to come from the health sector. Thus, the responsibility and blame for the poor performance of PHC, traceable to weak intersectoral actions, are often exclusively ascribed to a weak health sector. Secondly, PHC *per se* is not on the agenda of “nonhealth” sectors for their operational attention or targeting. Empirical evidence shows that PHC benefits from other sectors largely by coincidence. Thus, a decision to build a link road between communities is often driven by primary political goals, rather than core benefits such as enabling access to health care services and economic activities. Indeed, building such a road may lead to loss of crops and farmland without compensation, resulting in malnutrition and poverty in the affected households and communities. Thirdly, while ISC is presented in health policy documents such as Nigeria's National Health Policy [5] and in the academia, active collaborative projects between the health and “nonhealth” sectors are practically uncommon. The health sector itself does not seem to have sufficient practical initiatives towards ISC. This “tripod of neglect” is worsened by a “wait-and-see,” “let-us-hope-things-get-better” attitude from stakeholders. This is akin to a situation in which “everybody” was told to carry out a task (ISC for PHC), “somebody” (the health sector) was expected to do it; at the end, “nobody” did it!

To further illustrate this phenomenon, water-related diseases in Nigeria constitute about 80 per cent of the total disease burden [6]. While the provision of safe water is a key component of PHC and the MDGs, it is outside the statutory control of the health sector. It is commonplace in very poor communities that lack access to safe water for people to rely on contaminated water for survival, thus exposing them to the risk of diarrhoeal diseases. PHC *per se* is not on the agenda of the sector in charge of provision of safe water (e.g., works sector). Yet, intersectoral efforts to provide potable water for health are virtually unknown in some countries like Nigeria, despite repeated cholera epidemics in that country.

Furthermore, historical and contemporary health systems are largely structured around curative, promotive, preventive, and rehabilitative health services and a managerial support for them. On the field, the PHC system has been engaged in disease control programmes using integrated strategies such as health education, immunization, and drug campaigns. These arrangements have continued to be the framework for the assignment of roles, responsibilities and tasks to and within the health sector. But the system is currently ill-equipped and too conservative to optimally respond to the challenges posed by weak nonhealth inputs required for PHC. 

 In the circumstance, since the health sector has the mandate to implement PHC, the moral imperative appears to be on it to intervene in the impasse occasioned by the “tripod of neglect.”

## 6. Some Evidence Supporting Intersectoral Collaboration

The problem associated with ISC in developing countries lies with the scantiness of systematic programmatic experiences. This is probably different from what obtains in many developed countries where experiences in ISC may provide ample opportunity for empirical analysis, thus offering ready evidence for or against the utility of the strategy. The experiences shared in the paragraphs that follow are meant to justify the need to encourage the engagement of this strategy in the hope that further experience with time can provide additional evidence. 

In his study of political determinants of high mortality, morbidity, and disability rates related to emergency medical conditions, accidents, injuries and disasters in Nigeria, Aliyu initially conducted “content analysis of relevant documents, expert interviewing and consensus opinion.” Computer-aided risk analysis of his initial findings identified inadequate ISC between governmental sectors—including health, works and housing, defence and the police as well as the state and local governments—as a key determinant of the health problems. With the same software, he used policy mapping to generate recommendations which included a policy proposal that highlighted ISC as a process and an outcome objective and as a core component in policy implementation [7]. One of the values of this study is the emphatic placement of ISC as an essential requirement for improvement in health indices in Nigeria.

In a cross-sectional study carried out in a rural area in Bolivia, where Save the Children/US worked, the hypothesis that participation in intersectoral development programmes results in improved health behaviours and better health outcomes were tested. Four groups of 2,552 individuals in 499 households with varying levels of access to the organisation's programmes were compared, those participating in the health-only programmes; those with access to health and microenterprise credit, households participating in health and literacy programmes, those participating in all three programmes (health, credit and literacy), and a comparison group of households with no access to any of the programmes. The study showed that children in households participating in all three programmes were significantly less likely than children from comparison communities to be malnourished or at risk of becoming malnourished, even after controlling for such potentially confounding factors as social class, source of drinking water, and the availability of health facilities [8].

The major intersectoral approach in Nigeria's national response to the control of HIV/AIDS is a health sector initiative supervised by the Presidency. It includes a National Action Committee on HIV/AIDS (NACA) with membership drawn from the justice, social welfare, health, education, information, and other sectors. Similar bodies exist at state and local government area levels. These committees are statutorily headed by top government officials such as the state governors at the state levels, but functionally headed by the respective heads of the health sectors on their behalf. NACA has provided strong leadership for the control programme by engaging in advocacy to the private sector to sponsor HIV counselling and testing programmes for young people and economic support programmes for persons living with HIV/AIDS (PLWAs). NACA has also facilitated partnerships between local NGOs and foreign and international support agencies [9]. This way, a lot of intersectoral networks have developed (such as those between HIV/AIDS support groups and health facilities and between the Ministries of Health and Labour) to address the multifaceted challenges faced by PLWAs. 

 These studies and programmes provide some hope that ISC can reinforce PHC implementation and the rapid attainment of MDGs.

## 7. Recommended Roles for the Health Sector

From the foregoing, it is logical to expect that adequate ISC has the potential to bring about improvement in the health status of people. Indeed, as argued by Aliyu, adequate ISC—between the public and the private sector, and the government of the federation and private not-for-profit organisations, bilateral and multilateral institutions—is a key strategic initiative to reduce high mortality, morbidity, and disability rates related to emergency medical conditions, accidents, injuries, and disasters in Nigeria [7]. But there remains the question of *who* and *how* to kick-start or propel the process. This section addresses these issues.

The recommendations that follow (Box 1) are interventionist and, in some instances, deliberately unconventional for at least three reasons. First, the ineffectiveness and ultimate fatigue of collaboration and advocacy as currently practiced point to the need for a different set of approaches. Secondly, any initiative or process towards correcting the situation must have the potential to produce relatively early results by a short-chain process. Thirdly, the recommendations are based on the premise that the health sector, particularly its manpower and management, should take the frontal responsibility for the success of PHC.

The health sector should engage in intensive and sustained mobilisation of the political class (including the executive and legislature) and principal officers of relevant sectors through education and advocacies. These efforts should tactfully address their potential and actual contributions to the successes and failures of PHC, using striking examples. It is probably not satisfactory to hesitate in this regard based on the common assumption that “they know”. For example, the works sector should be educated using specific examples of how unmotorable roads cause delay in accessing emergency health services with the results of preventable but embarrassingly high disability and death rates in the country. The health sector should also organise relevant evidence-based workshops and seminars for the legislature to achieve informed and responsible legislative and oversight functions. In principle, support for and guideline on interventions like these are available in the literature [10, 11]. Such fora could be used to initiate discussions on how to accountably and fruitfully engage in collaborations.

The health sector should routinely design and present proposals for PHC-related infrastructures such as motorable roads and supply of potable water for implementation by the relevant “nonhealth” sectors. This should be followed up with sustained advocacies and mobilisation to gain acceptance and approval, inclusion into budgets and, sometimes, urgent implementation. Till now, the pattern in many countries is that respective nonhealth sectors propose and execute infrastructural projects without policy-directed inputs from the health sector. Again, the possible and understandable rejoinder from the health sector that “that is their job” should be minimised in the best interest of the public. Perhaps the health sector should draw strength from the fact that calls for ISC occasionally arise from other sectors too. For example, Kadiri opined that there was widespread agreement over the need to practically incorporate intersectoral approaches, including health and environmental concerns, into urban development [12].

Where funding of health-related projects in other sectors is a challenge, the health sector can intervene by mobilising for such funds on behalf of the relevant sector.

Again, health workers can engage in collective actions as pressure groups to demand for infrastructural development as an essential requirement for achieving PHC goals. That this is an ethical duty owed to the society is exemplified in Principle VII of the American Medical Association's Principles of Medical Ethics, which requires that physicians “recognise a responsibility to participate in activities contributing to an improved community” and to speak out on health care matters [13, 14].

Fortunately, the international community increasingly recognises the importance of ISC as a vital strategy towards better health for the poor and most vulnerable populations, especially with respect to interventions against those health determinants that are outside the control of the health sector [15, 16]. Health system experts in developing countries should take advantage of this realisation and of the familiar front of technical expertise presented by international aid agencies, and provide them with technical guidance. The guidance should be towards a responsible and exemplary management of programmes and projects using best practices in ISC.

Of particular interest is the opportunity provided for building local capacity through the transfer of appropriate and sustainable technology vis-à-vis the introduction of new equipment and techniques in health interventions. For example, the introduction of reuse prevention and needle-prick prevention features in syringes (through the Making Medical Injection Safer project) into some developing countries [17, 18] should be accompanied by an initiative to sustain its gain through the building of local capacity to manufacture these syringes. Such an effort will require collaboration between the health and manufacturing sectors, both of which, in turn, would collaborate with the relevant international agencies on patent and technology transfer issues, if necessary. Such collaboration may even extend to involve the educational sector for the training of relevant manpower. The entire process can be initiated, facilitated, and managed by the health sector.

Overall, the overarching proposal is the reconstruction, modeling, and institutionalisation of roles for the health sector beyond the facilitation and provision of conventional health services. One approach towards the implementation of these recommendations is to initiate pilot projects and studies that will guide the development of models, best practices, and frameworks. As experiences accumulate, evaluation will provide opportunity for improvement. Legal and policy frameworks, intensive and extensive capacity building to accountably take on the recommended roles and the creation of the required subsystems within the health system to manage ISC and other strategic linkages are required. Tasks and responsibilities should be specified to make the arrangement work. For example, as opined by McIntyre and Gilson with respect to South Africa, a health manager or team could be designated to signal when policies, programmes, and projects in other sectors, or the mismanagement of these, may conflict with efforts to promote health and health equity [19]. Thus, it is the build-up of experience that will provide incremental lessons and more supportive evidence for the utility of ISC. Neglecting to utilise ISC is unlikely to be a valuable option.

## 8. Conclusion

The health sector is generally expected to provide “health” to the public. The health sector, in turn, expects inputs from other sectors, many of which do not necessarily subscribe to the common purpose of or shared responsibility for health improvement. The stalemate created calls for intervention by the health sector itself—to take the initiative towards strong workable partnerships with other sectors locally and internationally. This would not only produce the possible benefits of intersectoral synergy, symbiosis, peer review, and efficiency (by avoiding effort duplication and wastage), but could also enhance the health status of the people. 

 Fortunately, Ministers of Health and representatives of the Ministries of Health from several developed and developing countries recently attended a strategic International Conference of Health for Development where they committed themselves to develop processes that will strengthen PHC. In particular, Articles 17, 22, and 24 in the resultant “Buenos Aires Declaration” highlight their commitment to engage in ISC and to even assist other sectors in developing health-related policies [20]. Beyond such and similar documents on health policies and health reform agendas in developing countries like Nigeria [5, 21], urgent actions are required to actualise ISC as a PHC strategy. Outcomes from the recommended pilot approach will provide opportunities for evolving best intersectoral practices with health sector initiative and evidence regarding its utility.

## Figures and Tables

**Figure 1:**
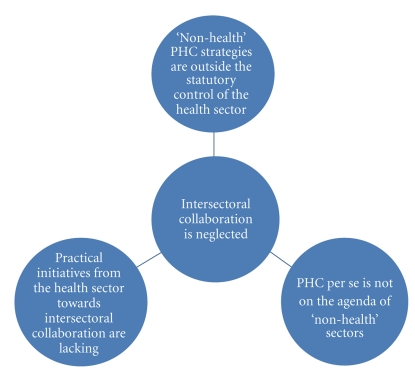
The “Tripod of Neglect” of intersectoral collaboration.

**Box 1:**
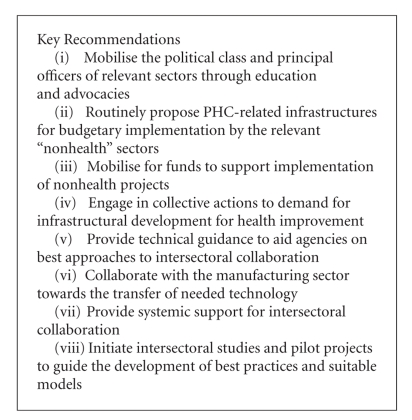
Key recommendations for the health sector.
